# Supplementary material for: Tuning charge transport dynamics via clustering of doping in organic semiconductor thin films
Source: Nat Commun. 2019 Jul 3;10:2827. doi: 10.1038/s41467-019-10567-5 (PMC6610129; doi:10.1038/s41467-019-10567-5)
Supplement: Supplementary file 1 — Supplementary Information [file 41467_2019_10567_MOESM1_ESM.pdf]

Supplemental Information:

Tuning charge transport dynamics via clustering of doping in organic semiconductor thin films

Connor J. Boyle,[1] Meenakshi Upadhyaya,[2] Peijian Wang,[3] Lawrence A. Renna,[1] Michael Lu-Díaz,[1] Seung Pyo Jeong,[1] Nicholas Hight-Huf,[1] Ljiljana Korugic-Karasz,[4] Michael D. Barnes,[1,3] Zlatan Aksamija,[2,*] D. Venkataraman[1,*]

[1]Department of Chemistry, University of Massachusetts Amherst, Amherst, MA, USA

[2]Department of Electrical and Computer Engineering, University of Massachusetts Amherst, Amherst, MA, USA

[3]Department of Physics, University of Massachusetts Amherst, Amherst, MA, USA

[4]Department of Polymer Science and Engineering, University of Massachusetts Amherst, Amherst, MA, USA

[*]e-mail: dv@chem.umass.edu, zlatana@umass.edu

Contents

**Supplementary Methods**	4
**Supplementary Figure 1:** The effect of annealing PDPP4T at 75 °C on its charge transport profile. The PDPP4T annealed at 75 °C and then doped at 25 °C (gray diamonds) has a charge transport profile that resembles PDPP4T doped at 25 °C (black hexagons) more so than PDPP4T doped at 75 °C (red circles), indicating that changes in the charge transport profile by changing the doping temperature cannot be explained by thermal annealing alone.	5
**Supplementary Figure 2:** The effect of repeatedly doping, dedoping, and redoping the same film of PDPP4T. The sample of PDPP4T that was doped at 25 °C and characterized as it spontaneously dedoped (black circles), was next redoped and characterized the same way (gray squares), and finally redoped a third time and characterized yet again (gray triangles). The same charge transport trend is recovered with each repetition and does not resemble that of PDPP4T doped at 75 °C (red lines).	5
**Supplementary Figure 3:** Log-log plot of the α vs. σ for PDPP4T, prepared in-house with M_w = 320 kDa and $Đ$ = 4.4, doped at 25 °C (black upward triangles) and 75 °C (red downward triangles).	6
**Supplementary Figure 4:** The log-log plot of measured Seebeck coefficient vs. conductivity and fits to Snyder and Kang's charge transport model for **a** P3HT doped at 25 °C (black circles), **b** P3HT doped at 75 °C (red squares), **c** PDPP4T doped at 25 °C (blue upward triangles), and **d** PDPP4T doped at 75 °C (purple downward triangles). The best fit (colored solid curves) is compared to the s = 1 (gray solid curves) and s = 3 (gray dashed curves).	7-8
**Supplementary Figure 5:** Photoluminescence microscopy images of **a** a pristine P3HT film indicating intense, uniform photoluminescence, **b** an iodine-doped (at 25 °C) P3HT film indicating quenched, non-uniform photoluminescence, and **c** the PL emission spectra of the pristine P3HT (red), a lightly doped patch from the iodine doped P3HT (maroon), and a densely doped patch of the iodine doped P3HT (black). Exposure time: 0.2 s.	9
**Supplementary Figure 6:** Log-log plot of Seebeck coefficient vs. conductivity computed for **a** Gaussian, **b** doping induced heavy tailed and **c** clustering induced heavy tailed DOS is fit with Snyder and Kang's charge transport model. The doping induced distribution is computed with dopant concentration N_d = 10% and cluster concentration C_s = 1, and the clustering induced distribution with N_d = 3% and C_s = 3.	10
**Supplementary Figure 7**: 1-D wide angle X-ray scattering patterns of (a) P3HT films before/after annealing at 75 °C, and (b) PDPP4T films before/after annealing at 75 °C. The P3HT or PDPP4T film was annealed at 75 °C for 2 h. The same P3HT or PDPP4T film was used before/after annealing for comparison.	11
**Supplementary Figure 8**: 1-D wide angle X-ray scattering patterns of (a) P3HT films doped at 25 °C, and after dedoping, (b) P3HT films doped at 75 °C, and after dedoping, (c) PDPP4T films doped at 25 °C, and after dedoping, and (d) PDPP4T films doped at 25 °C, and after dedoping. The wide angle X-ray scattering patterns of the pristine polymer are also provided for comparison.	12

**Supplementary Note 1: Simulation Details**	13
**Supplementary Note 2: Modified Gaussian Model and the Transport Parameter**	13
**Supplementary References**	14

Supplementary Methods

Effects of annealing, redoping, and source of PDPP4T

Annealing was performed by placing the PDPP4T film into a 75 °C oven for 2 h. This film was then immediately transferred to a 25 °C 20 mL vial containing a 1 mL vial with 50±5 mg iodine for 2 h of doping before measuring the conductivity and Seebeck coefficient, as shown in **supplementary Figure 1**.

Each time PDPP4T was redoped, redoping was performed by removing the PDPP4T film from the conductivity and Seebeck coefficient measurement apparatus and immediately transferring it to a 25 °C 20 mL vial containing a 1 mL vial with 50±5 mg iodine for 2 h before measuring the conductivity and Seebeck coefficient of this film again, as shown in **supplementary Figure 2**.

The effects of annealing PDPP4T and redoping PDPP4T were studied using the same PDPP4T described in the main text, sourced from Ossila, (M_w = 171,138 Da, $Đ$ = 2.45). To verify the doping temperature-dependence of the α vs. σ curve, PDPP4T was prepared in-house according to the established procedures.[1] The M_n was 72.8 kDa and the $Đ$ was 4.4, as determined by an Agilent Gel Permeation Chromatography system with refractive index detector using chloroform as the eluent and polystyrene as a standard. Each home-made PDPP4T film was prepared the same way as commercially sourced PDPP4T films: by dropcasting onto a 45 °C preheated, 1.1 cm × 2.2 cm glass coverslip from an 8 mg/mL chloroform solution, covering immediately with a watch glass for 10 minutes at 45 °C, and finally allowing to dry under ambient conditions for no less than 24 h.

Fitting to the charge transport model

Using the transport model $\sigma_{\mathrm{E}}(E,T) = \sigma_{\mathrm{E}_0}(T)\left(\frac{E-E_{\mathrm{t}}}{k_{\mathrm{B}}T}\right)^{s}$, $\sigma = \int \sigma_{\mathrm{E}}\left(-\frac{\partial f}{\partial E}\right)\mathrm{d}E$, and $\alpha = \left(\frac{k_B}{q}\right)\int\left(\frac{E-E_{\mathrm{F}}}{k_{\mathrm{B}}T}\right)\frac{\sigma(E)}{\sigma}dE$ and a reduced Fermi level, $\eta = (E_{\mathrm{F}} - E_{\mathrm{t}})/k_{\mathrm{B}}T$,[2] a reduced Fermi level was initially found for each experimentally measured α using a root finding algorithm. Next, σ_{E_0} was determined using a least squares regression fit to the charge transport model of experimentally measured σ vs. calculated η. The best fit for s = 1, s = 3, and best fit overall for a positive half-integer or integer s are shown in **supplementary Figure 4**.

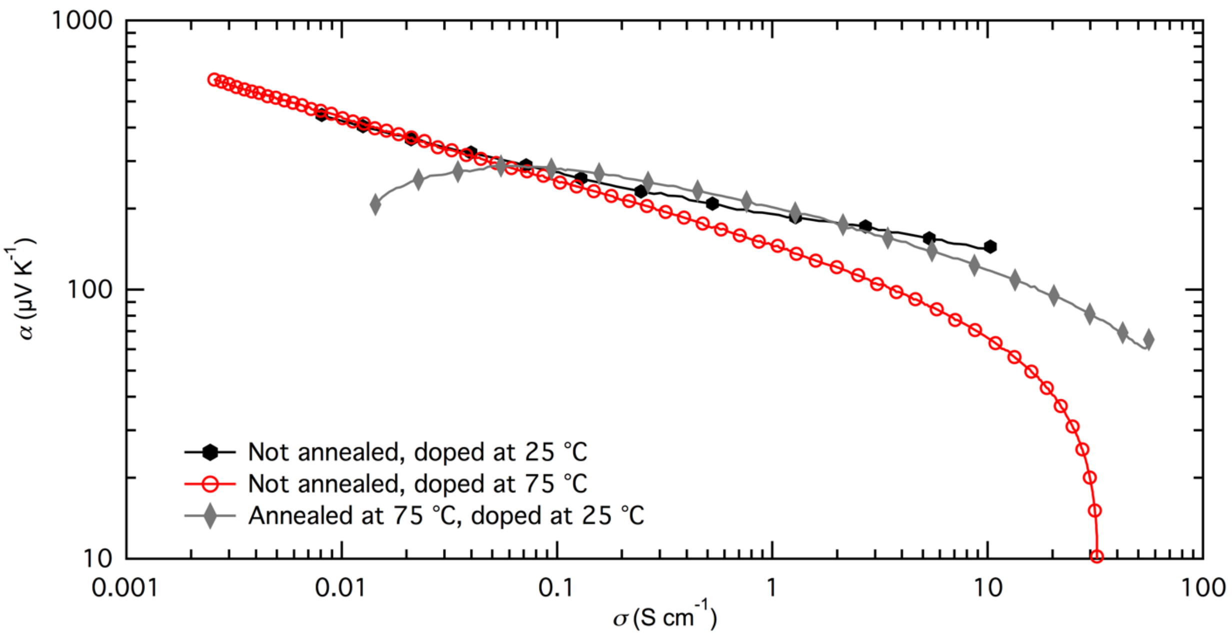

Supplementary Figure 1: The effect of annealing PDPP4T at 75 °C on its charge transport profile. The PDPP4T annealed at 75 °C and then doped at 25 °C (gray diamonds) has a charge transport profile that resembles PDPP4T doped at 25 °C (black hexagons) more so than PDPP4T doped at 75 °C (red circles), indicating that changes in the charge transport profile by changing the doping temperature cannot be explained by thermal annealing alone.

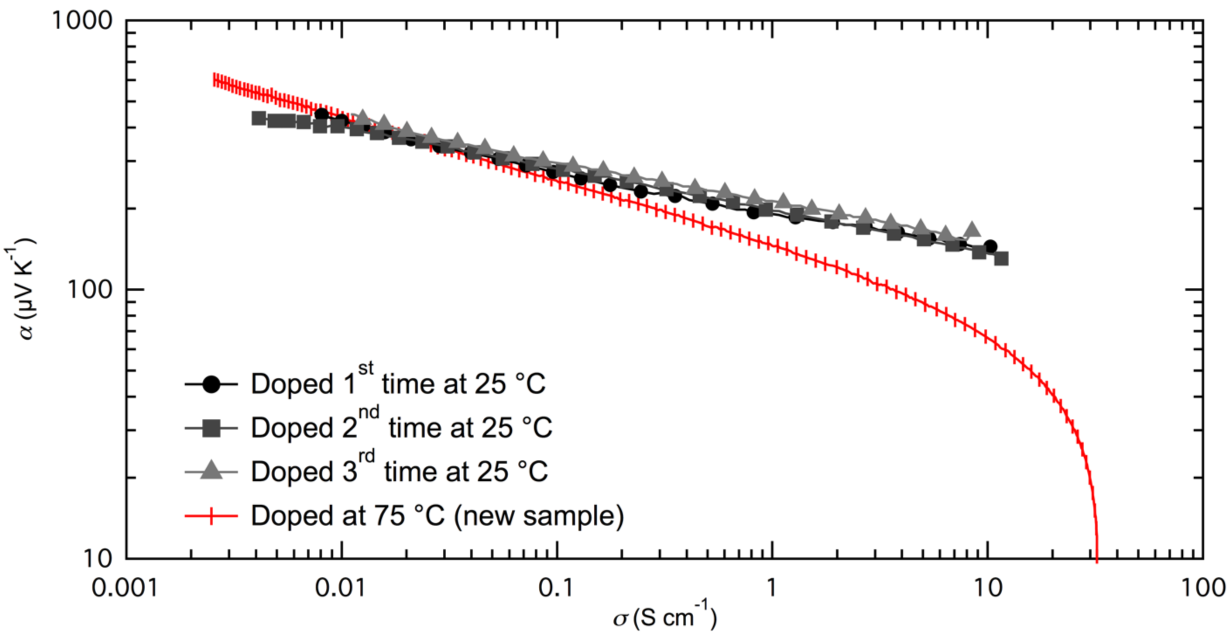

Supplementary Figure 2: The effect of repeatedly doping, dedoping, and redoping the same film of PDPP4T. The sample of PDPP4T that was doped at 25 °C and characterized as it spontaneously dedoped (black circles), was next redoped and characterized the same way (gray squares), and finally redoped a third time and characterized yet again (gray triangles). The same charge transport trend is recovered with each repetition and does not resemble that of PDPP4T doped at 75 °C (red lines).

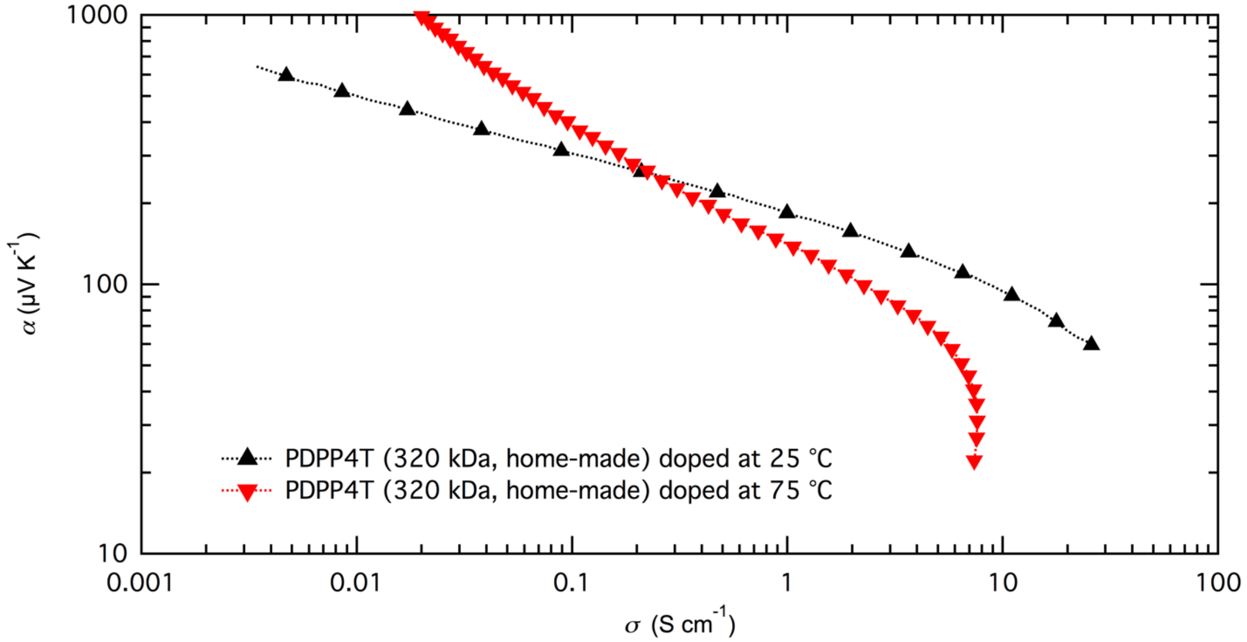

Supplementary Figure 3: Log-log plot of the α vs. σ for PDPP4T, prepared in-house with M_w = 320 kDa and $Đ$ = 4.4, doped at 25 °C (black upward triangles) and 75 °C (red downward triangles).

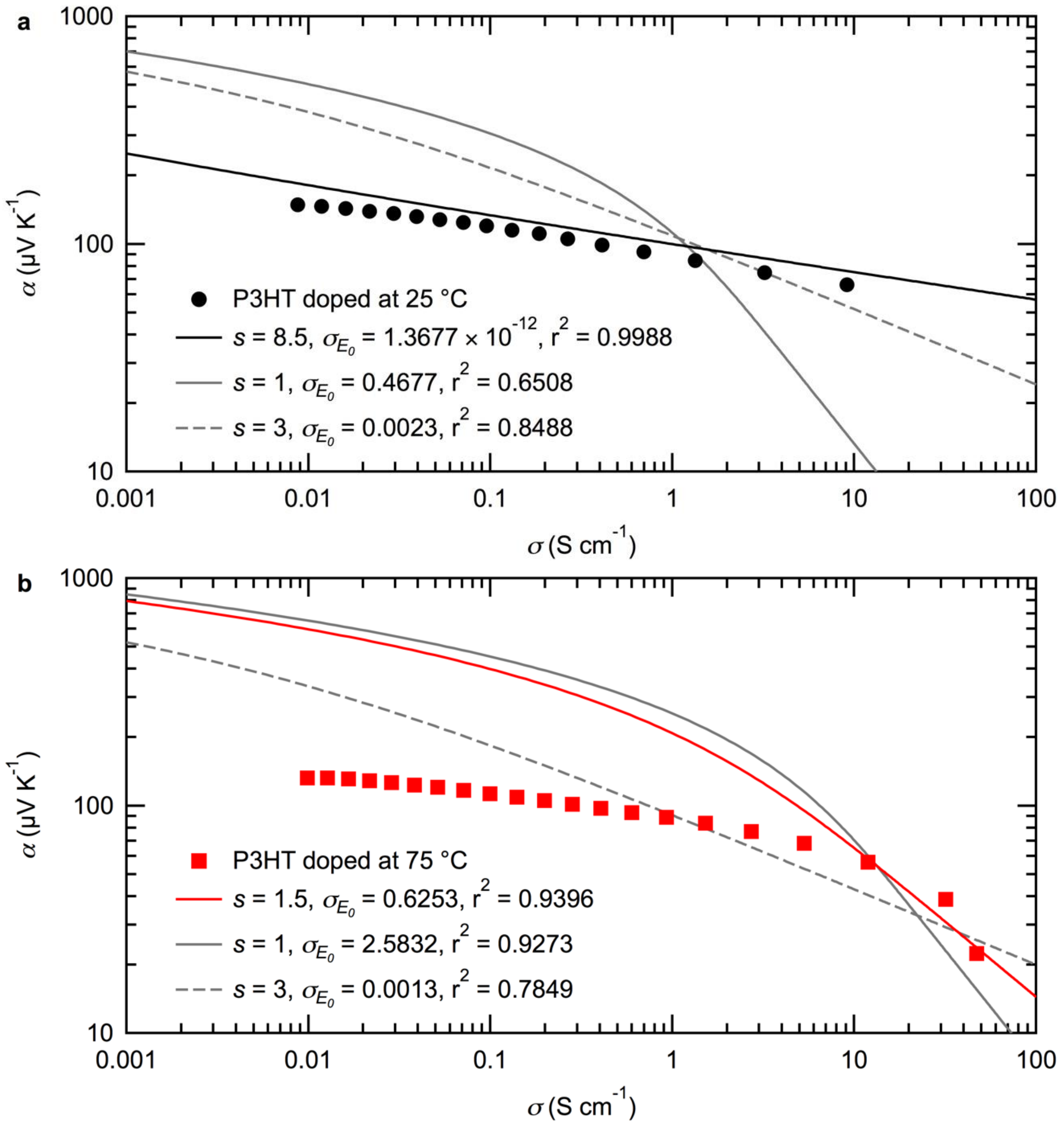

Supplementary Figure 4, ***Continued on the next page.***

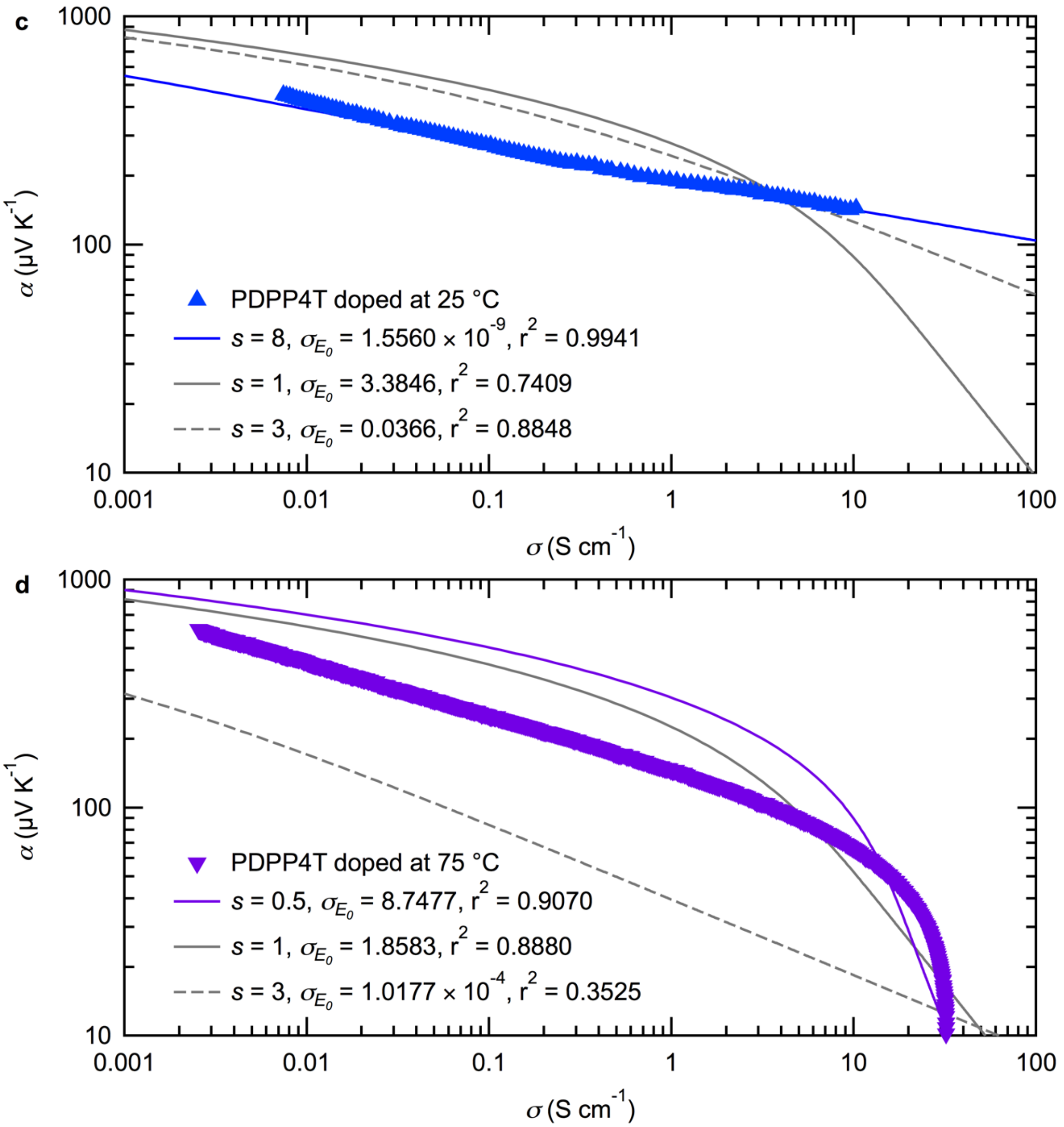

Supplementary Figure 4: The log-log plot of measured Seebeck coefficient vs. conductivity and fits to Snyder and Kang's charge transport model for **a** P3HT doped at 25 °C (black circles), **b** P3HT doped at 75 °C (red squares), **c** PDPP4T doped at 25 °C (blue upward triangles), and **d** PDPP4T doped at 75 °C (purple downward triangles). The best fit (colored solid curves) is compared to the s = 1 (gray solid curves) and s = 3 (gray dashed curves).

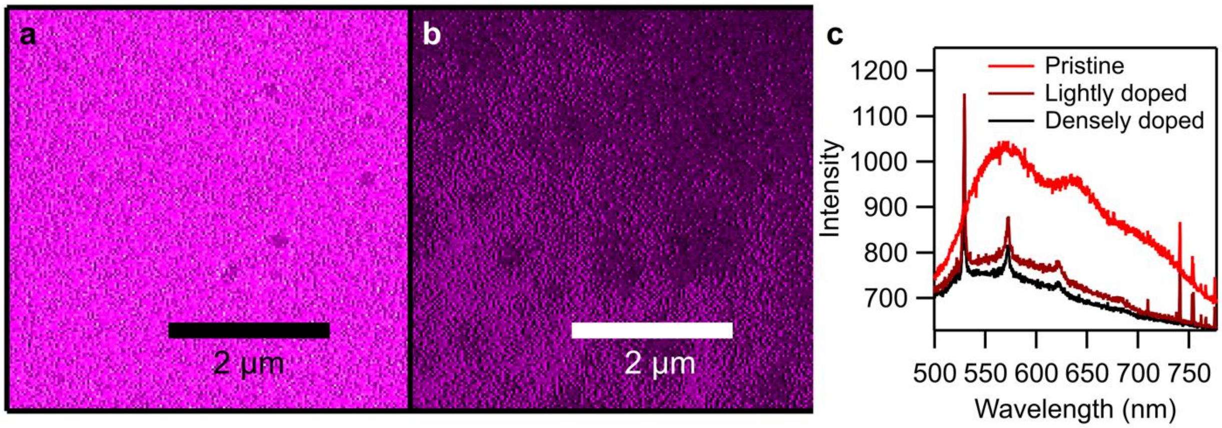

Supplementary Figure 5: Photoluminescence microscopy images of **a** a pristine P3HT film indicating intense, uniform photoluminescence, **b** an iodine-doped (at 25 °C) P3HT film indicating quenched, non-uniform photoluminescence, and **c** the PL emission spectra of the pristine P3HT (red), a lightly doped patch from the iodine doped P3HT (maroon), and a densely doped patch of the iodine doped P3HT (black). Exposure time: 0.2 s.

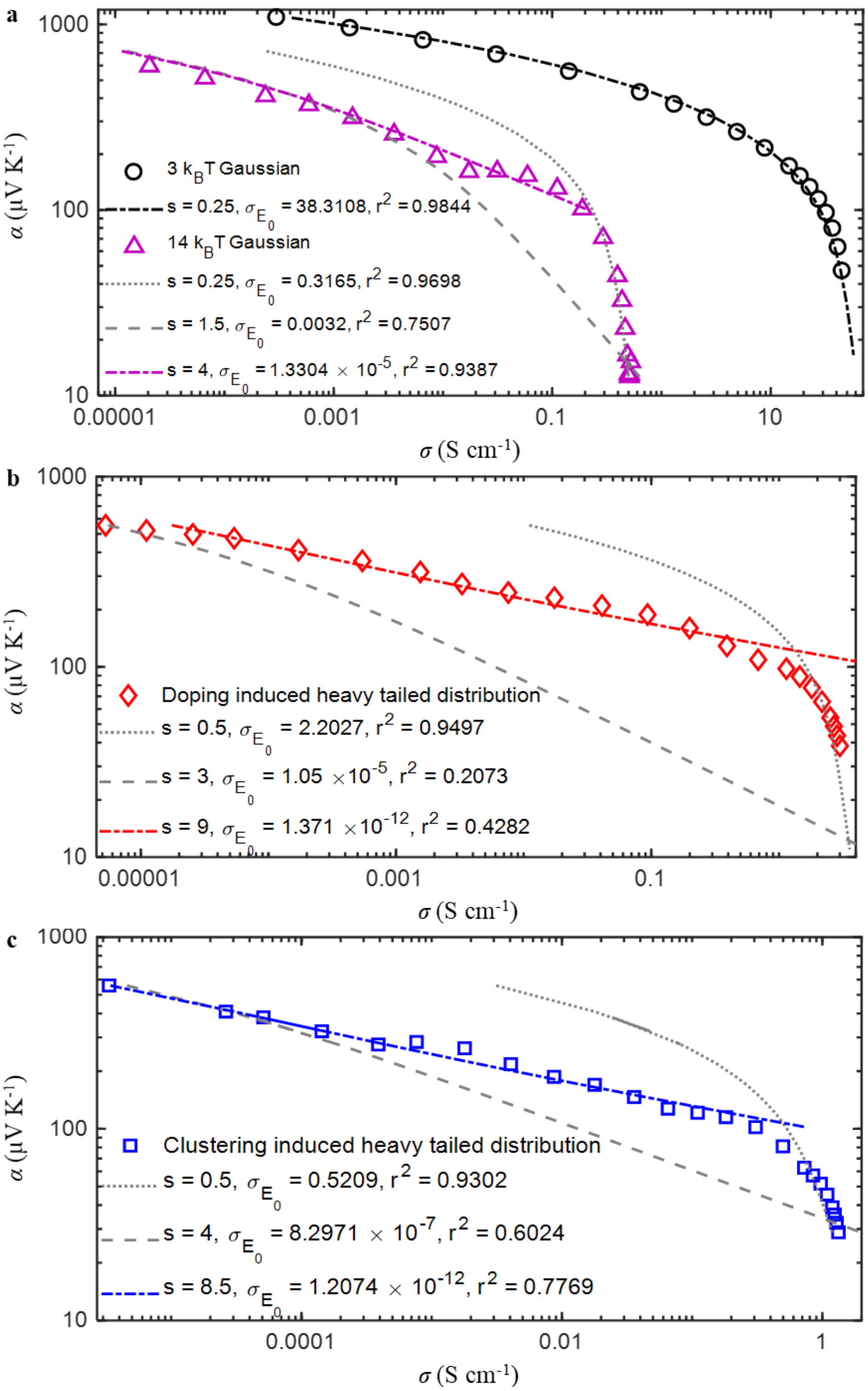

Supplementary Figure 6: Log-log plot of Seebeck coefficient vs. conductivity computed for **a** Gaussian, **b** doping induced heavy tailed and **c** clustering induced heavy tailed DOS is fit with Snyder and Kang's charge transport model. The doping induced distribution is computed with dopant concentration N_d = 10% and cluster concentration C_s = 1, and the clustering induced distribution with N_d = 3% and C_s = 3.

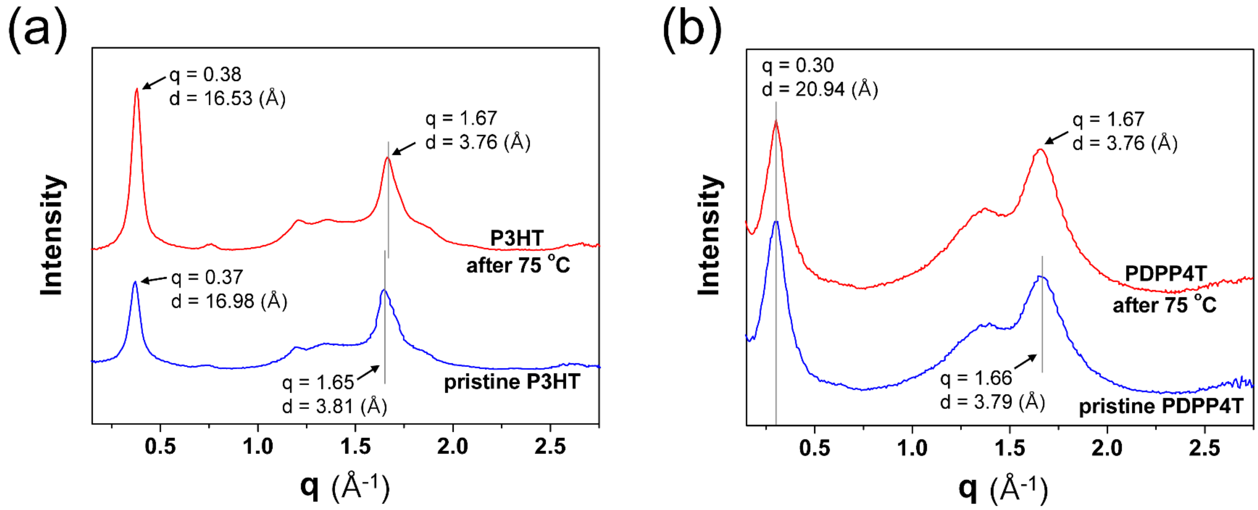

Supplementary Figure 7: 1-D wide angle X-ray scattering patterns of (a) P3HT films before/after annealing at 75 °C, and (b) PDPP4T films before/after annealing at 75 °C. The P3HT or PDPP4T film was annealed at 75 °C for 2 h. The same P3HT or PDPP4T film was used before/after annealing for comparison.

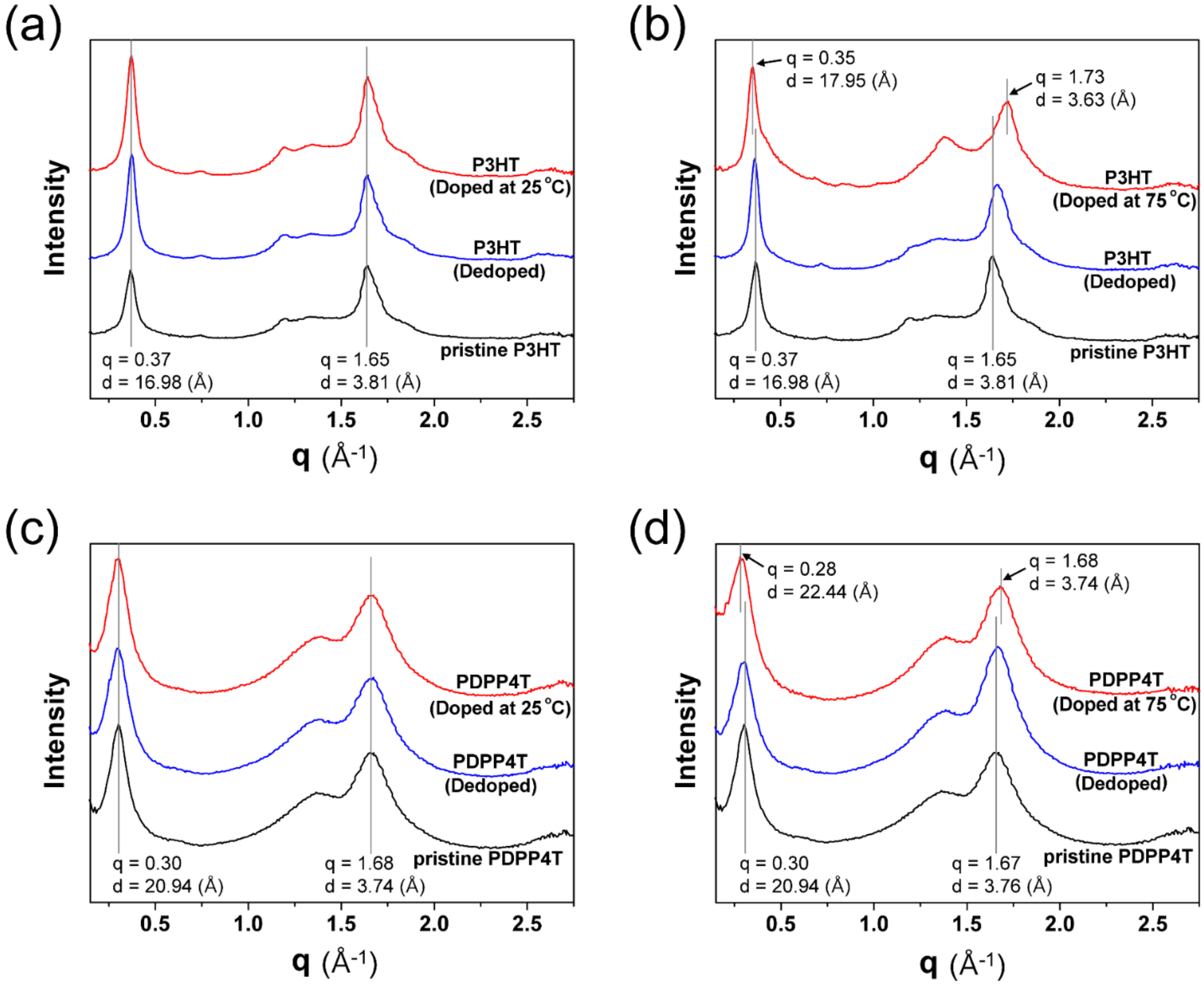

Supplementary Figure 8: 1-D wide angle X-ray scattering patterns of (a) P3HT films doped at 25 °C, and after dedoping, (b) P3HT films doped at 75 °C, and after dedoping, (c) PDPP4T films doped at 25 °C, and after dedoping, and (d) PDPP4T films doped at 75 °C, and after dedoping. The wide angle X-ray scattering patterns of the pristine polymer are also provided for comparison.

Supplementary Note 1: Simulation Details

Solving the non-linear PME

We solve the non-linear PME using a standard iterative non-linear solver. First, we cast the PME as zero-finding for a system of equations $F_i(p) = \sum_j \left[W_{ij} p_i \left(1 - p_j\right) - W_{ji} p_j (1 - p_i)\right] = 0$, which can be written in terms of the in- and out-scattering as $F_i(p) = p_i S_{\text{out}}(p) - (1 - p_i) S_{\text{in}}(p)$, where $S_{\text{out}}(p) = \sum_j \left[W_{ij} p_i \left(1 - p_j\right)\right]$ and $S_{\text{in}}(p) = \sum_j \left[W_{ji} p_j\right]$. Since both in- and out-scattering terms depend on the unknown p, $F_i(p)$ is nonlinear and a fixed-point iteration can stall, resulting in poor convergence for some cases. Hence, we follow a fixed-point iteration for the p_i such that $p_i^{n+1} = S_{in}(p^n)/[S_{\text{in}}(p^n) + S_{\text{out}}(p^n)]$ with the initial p_i^0 being the Fermi-Dirac distribution, only for the first few iterations and then use the resulting estimate of p_i as an initial guess where we numerically solve for $F_i(p)$. Rather than solving for the site occupancies p_i, we solve for their deviation away from equilibrium $\Delta p_i = p_i - p_i^0$. Combining this with $0 = \Sigma_j [W_{ij} p_i \left(1 - p_j\right) - W_{ji} p_j (1 - p_i)]$ and simplifying we get $F_i(p) = \Delta p_i S_{\text{out}}(p) - (1 - p_i^0) S_{\text{in}}(p) = 0$.

We arrange the 35×25×25 array of Δp_i's into a column vector p and compute the Jacobian matrix of derivatives of F_i with respect to p_j as $J_{ij} = dF_i/dP_j = -W_{ji}(1 - p_i)$. Then we apply the Levenberg-Marquardt algorithm,[3] as implemented in MATLAB's fsolve function, with the known Jacobian matrix, which requires a linear solve at each iteration but typically converges in a few iterations due to its high rate of convergence. The linear solver is a preconditioned Conjugate Gradients algorithm with a banded preconditioner based on an incomplete Cholesky factorization.

Supplementary Note 2: Modified Gaussian Model and the Transport Parameter

We plot the simulated α vs. σ for Gaussian and heavy tailed DOS and try to find the best fit values of transport parameter *s* from the Kang and Snyder's charge transport model. A narrow Gaussian DOS (<6$k_B T$) fits *s* = 0.5 very closely (**supplementary Figure 6a)**, however, with increasing energetic disorder (wide Gaussian) and with dopant clustering (heavy tailed distribution), it is not possible to fit the entire curve with a single value of *s* (**supplementary Figure 6).** Parts of the α vs. σ curve can be fit with different *s* values and we find the best fit for the flat part of the curve (nearly constant α) to show the effect of the exponentially decaying tail of the DOS leading to high values of *s*. The fact that a single value of the transport parameter *s* cannot always fit the entire α vs. σ curve shows the limitations of a band model in capturing the effect of shape and size of the DOS on charge transport in highly disordered systems.

Supplementary References:

1. Li, Y. et al. Annealing-free high-mobility diketopyrrolopyrrole-quaterthiophene copolymer for solution-processed organic thin film transistors. *J. Am. Chem. Soc.* **133**, 2198–2204 (2011).

2. Kang, S. D. & Snyder, G. J. Charge-transport model for conducting polymers. *Nat. Mater.* **16**, 252–257 (2017).

1 Li, Y. *et al.* Annealing-free high-mobility diketopyrrolopyrrole-quaterthiophene copolymer for solution-processed organic thin film transistors. *J. Am. Chem. Soc.* **133**, 2198–2204 (2011).

2 Kang, S. D. & Snyder, G. J. Charge-transport model for conducting polymers. *Nat. Mater.* **16**, 252–257 (2017).

3 Press, W. H. *et al*. Numerical Recipes in C (2nd Ed.): The Art of Scientific Computing. Cambridge University Press, New York, NY, USA (1992).
